# Blood-based epigenetic estimators of chronological age in human adults using DNA methylation data from the Illumina MethylationEPIC array

**DOI:** 10.1186/s12864-020-07168-8

**Published:** 2020-10-27

**Authors:** Yunsung Lee, Kristine L. Haftorn, William R. P. Denault, Haakon E. Nustad, Christian M. Page, Robert Lyle, Sindre Lee-Ødegård, Gunn-Helen Moen, Rashmi B. Prasad, Leif C. Groop, Line Sletner, Christine Sommer, Maria C. Magnus, Håkon K. Gjessing, Jennifer R. Harris, Per Magnus, Siri E. Håberg, Astanand Jugessur, Jon Bohlin

**Affiliations:** 1 Department of Genetics and Bioinformatics, Norwegian Institute of Public Health, Oslo, Norway; 2 Institute of Health and Society, Faculty of Medicine, University of Oslo, Oslo, Norway; 3 Centre for Fertility and Health, Norwegian Institute of Public Health, Oslo, Norway; 4 Department of Global Public Health and Primary Care, University of Bergen, N-5020 Bergen, Norway; 5 Deepinsight, Karl Johans gate 8, Oslo, Norway; 6 Oslo Centre for Biostatistics and Epidemiology, Section for Research Support, Oslo University Hospital, Oslo, Norway; 7 Department of Medical Genetics, Oslo University Hospital, Oslo, Norway; 8 PharmaTox Strategic Research Initiative, School of Pharmacy, Faculty of Mathematics and Natural Sciences, University of Oslo, Oslo, Norway; 9 Department of Internal Medicine, Akershus University Hospital, Kongsvinger, Norway; 10 Department of transplantation medicine, Institute of Clinical medicine, University of Oslo, Oslo, Norway; 11 Institute of Clinical Medicine, Faculty of Medicine, University of Oslo, Oslo, Norway; 12 The University of Queensland Diamantina Institute, University of Queensland, Woolloongabba, QLD 4102 Australia; 13 K.G. Jebsen Center for Genetic Epidemiology, Department of Public Health and Nursing, Norwegian University of Science and Technology, Trondheim, Norway; 14 Population Health Science, Bristol Medical School, University of Bristol, Bristol, UK; 15 Department of Clinical Sciences, Clinical Research Centre, Lund University, Malmö, Sweden; 16 Finnish Institute of Molecular Medicine, Helsinki University, Helsinki, Finland; 17 Department of Pediatric and Adolescents Medicine, Akershus University Hospital, Lørenskog, Norway; 18 Institute of Clinical Medicine, University of Oslo, Campus AHUS, Lørenskog, Norway; 19 Department of Endocrinology, Morbid Obesity and Preventive Medicine, Oslo University Hospital, Oslo, Norway; 20 MRC Integrative Epidemiology Unit at the University of Bristol, Bristol, UK; 21 Division for Infection Control and Environmental Health, Department of Infectious Disease Epidemiology and Modelling, Norwegian Institute of Public Health, Oslo, Norway

**Keywords:** DNA methylation, Epigenetic age, Chronological age, Illumina MethylationEPIC BeadChip, MoBa

## Abstract

**Background:**

Epigenetic clocks have been recognized for their precise prediction of chronological age, age-related diseases, and all-cause mortality. Existing epigenetic clocks are based on CpGs from the Illumina HumanMethylation450 BeadChip (450 K) which has now been replaced by the latest platform, Illumina MethylationEPIC BeadChip (EPIC). Thus, it remains unclear to what extent EPIC contributes to increased precision and accuracy in the prediction of chronological age.

**Results:**

We developed three blood-based epigenetic clocks for human adults using EPIC-based DNA methylation (DNAm) data from the Norwegian Mother, Father and Child Cohort Study (MoBa) and the Gene Expression Omnibus (GEO) public repository: 1) an Adult Blood-based EPIC Clock (ABEC) trained on DNAm data from MoBa (*n* = 1592, age-span: 19 to 59 years), 2) an extended ABEC (eABEC) trained on DNAm data from MoBa and GEO (*n* = 2227, age-span: 18 to 88 years), and 3) a common ABEC (cABEC) trained on the same training set as eABEC but restricted to CpGs common to 450 K and EPIC. Our clocks showed high precision (Pearson correlation between chronological and epigenetic age (r) > 0.94) in independent cohorts, including GSE111165 (*n* = 15), GSE115278 (*n* = 108), GSE132203 (*n* = 795), and the Epigenetics in Pregnancy (EPIPREG) study of the STORK Groruddalen Cohort (*n* = 470). This high precision is unlikely due to the use of EPIC, but rather due to the large sample size of the training set.

**Conclusions:**

Our ABECs predicted adults’ chronological age precisely in independent cohorts. As EPIC is now the dominant platform for measuring DNAm, these clocks will be useful in further predictions of chronological age, age-related diseases, and mortality.

**Supplementary Information:**

The online version contains supplementary material available at 10.1186/s12864-020-07168-8.

## Background

Aging is a biological phenomenon that is characterized by reduced functional capacity [1, 2]. Because chronological age is an imperfect surrogate of aging [3–6], the concept of biological aging that can capture the different rate of functional deterioration across individuals has been suggested [1]. Given the significance of biological aging, a variety of predictors of biological age have been constructed based on known hallmarks of aging [6, 7], including telomere length [8], metabolic rate [9], DNA methylation (DNAm) [10], CD4+ and CD8+ T cell ratio [11], proteomic alterations [12], and gut microbiota [13]. Among these, DNAm-based estimators of chronological age (referred to as epigenetic clocks) have garnered the most interest due to their remarkable precision in estimating chronological age, age-related diseases, and all-cause mortality [4, 14–18].

Epigenetic age is a linear combination of DNAm levels at specific CpGs, which are weighted by their respective coefficients estimated through an epigenetic clock. Most of the previously published epigenetic clocks (the Hannum Blood-based clock [19], Horvath Pan-tissue clock [20], Levine PhenoAge clock [16], and Horvath Skin & Blood clock [3]) were based on specific CpGs from the Illumina HumanMethylation450 BeadChip (450 K). This platform has recently been replaced by the Illumina MethylationEPIC BeadChip (EPIC). EPIC is a major improvement over its predecessor, 450 K (> 450,000 CpGs), in terms of the number of probes (> 850,000 CpGs) and the genomic coverage of regulatory elements [21]. To our knowledge, only one EPIC-based epigenetic clock has been published (the Alsaleh EPIC clock [22]). This clock was trained on a relatively small training set and was not sufficiently validated in independent cohorts. Thus, it remains unclear to what extent EPIC contributes to increased precision and accuracy in the prediction of chronological age.

We developed three blood-based epigenetic clocks for human adults: 1) an Adult Blood-based EPIC Clock (ABEC) trained on EPIC-derived DNAm data from adult peripheral blood in a sub-study of the Norwegian Mother, Father and Child Cohort Study (MoBa) [23] called the STudy of Assisted Reproductive Technology (MoBa-START); 2) an extended ABEC (eABEC) trained on MoBa-START and publicly available DNAm data from the Gene Expression Omnibus (GEO) with the aim of improving the performance of ABEC; and 3) a common ABEC (cABEC) trained on the same training set as eABEC but restricted to CpGs common to 450 K and EPIC. The purpose of cABEC was to determine whether the additional CpGs on EPIC improved predictions of chronological age. We validated our clocks and the other published clocks (the Hannum Blood-based clock, Horvath Pan-tissue clock, Levine PhenoAge clock, Horvath Skin & Blood clock, Alsaleh EPIC clock, and Zhang clock) in EPIC-derived DNAm data from independent cohorts, including publicly available DNAm data from GEO and the Epigenetics in Pregnancy (EPIPREG) study of the STORK Groruddalen Cohort (STORK) [24].

## Results

### Peripheral blood-based DNA methylation

We trained an epigenetic clock using elastic net regression on DNAm data from 1592 adults who were mothers and fathers in MoBa-START (796 women and 796 men). The chronological age of these adults ranged from 19 to 59 years (19 to 46 years for women and 19 to 59 years for men). DNAm on these individuals was measured using EPIC. For the current analyses, we focused on the 770,586 autosomal CpGs that remained after quality control (see Methods). Table 1 provides additional details regarding the MoBa-START samples.

**Table 1:** Description of the peripheral whole-blood-derived DNAm data on the EPIC platform

Cohort	Tissue type	Platform	GEO submitter	N	Normalization Method^a^	Probe exclusion Criteria^b^	Age range (years)
**ABEC**							
**Training data**							
MoBa-START	Peripheral whole blood	EPIC	–	1592	BMIQ	SC, CH, DP, SNP	19–59
**Test data**							
MoBa-START	Peripheral whole blood	EPIC	–	424	BMIQ	SC, CH, DP, SNP	20–58
**eABEC**							
**Training data**							
MoBa-START	Peripheral whole blood	EPIC	–	1592	BMIQ	SC, CH, DP, SNP	19–59
GSE116339	Peripheral whole blood	EPIC	Curtis et al. [25]	635	Noob	SC	23–88
**Test data**							
MoBa-START	Peripheral whole blood	EPIC	–	424	BMIQ	SC, CH, DP, SNP	20–58
GSE111165	Peripheral whole blood	EPIC	Shinozaki et al. [26]	15	Noob	SC	24–61
GSE115278	Peripheral whole blood	EPIC	Arpon et al. [27]	108	Noob	SC	19–66
**Other test data**							
EPIPREG	Peripheral whole blood	EPIC	–	470	FunNorm	SC, CH, DP, SNP	19–42
GSE132203	Peripheral whole blood	EPIC	Kilaru et al. [28]	795	Noob	SC	18–76

^a^ Pre-processing method for quantifying DNAm levels in the range of 0 to 1

*Noob* Normal-exponential out-of-band [29]

*BMIQ* Beta-mixture quantile dilation [30]

*FunNorm* Functional normalization [31]

^b^ Probe exclusion criteria

*SC* Sex chromosome, *CH* cross-hybridizing, *DP* detection *P*-value < 0.01 and *SNP* single-nucleotide polymorphism

### Adult blood-based EPIC clock (ABEC)

**Fig. 1:**
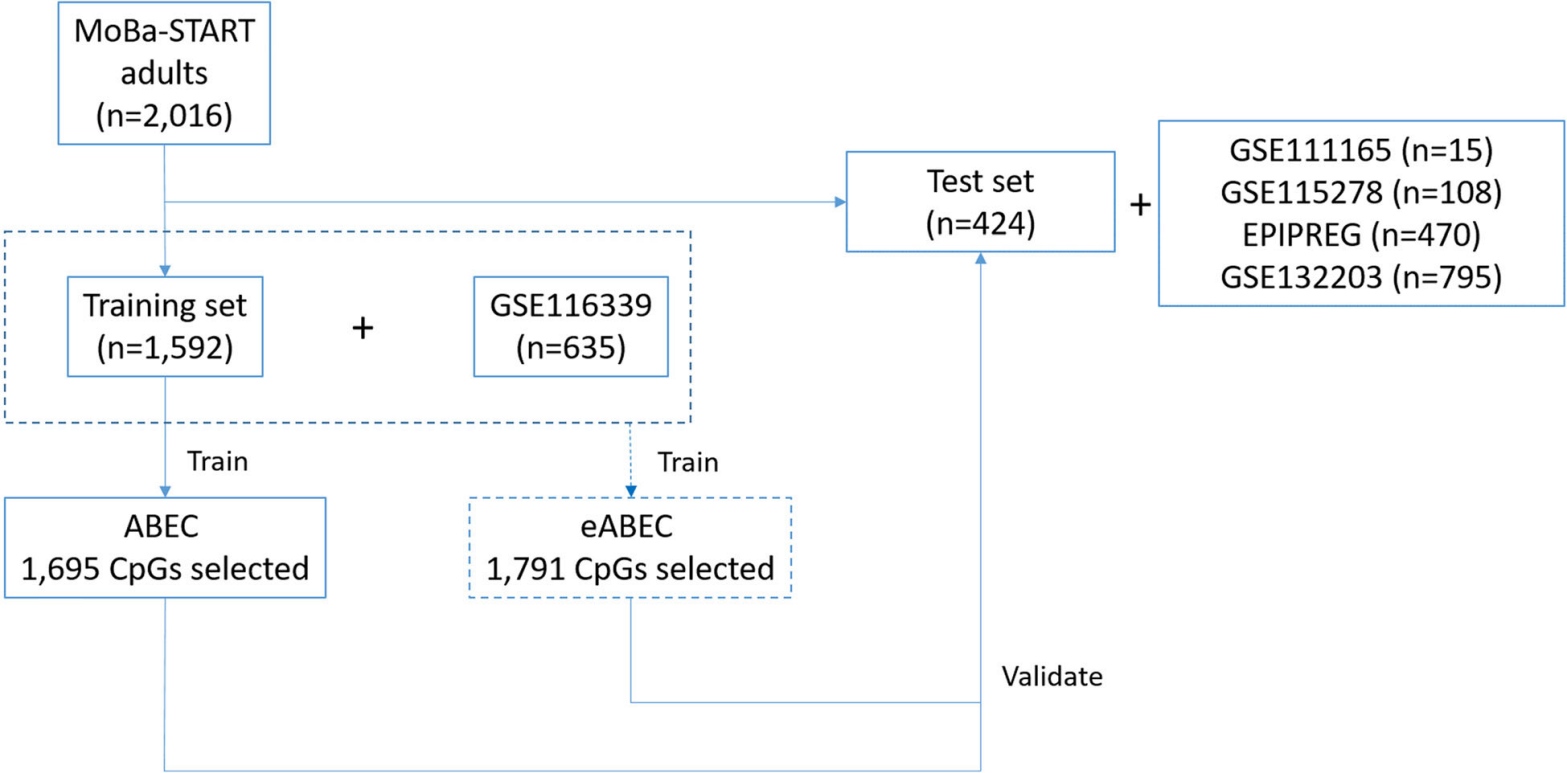
Analysis flow. MoBa-START adults were randomly assigned to a training and a test set

Figure 1 summarizes our analysis flow.

We developed ABEC using a blood-based DNAm dataset consisting of adults (training set *n* = 1592, Table 1, Fig. 1). We used elastic net regression [32] to select the most predictive CpGs for chronological age. The resulting regression comprised 1695 CpGs. The predicted DNAm age was calculated using the following equation:

$$ DNAm\  Ag{e}_j={\hat{\beta}}_{(Intercept)}+{X}_{cg1,j}{\hat{\beta}}_{cg1}+{X}_{cg2,j}{\hat{\beta}}_{cg2}+\dots +{X}_{cg1695,j}{\hat{\beta}}_{cg1695}, $$

where *DNAm Age*_*j*_ is the epigenetic age of the *j* th individual, and *X*_*cgi*, *j*_ refers to the DNAm level of the *j* th individual at the *i* th CpG site. The estimated intercept and beta coefficients are provided in Supplementary File 1.

**Fig. 2:**
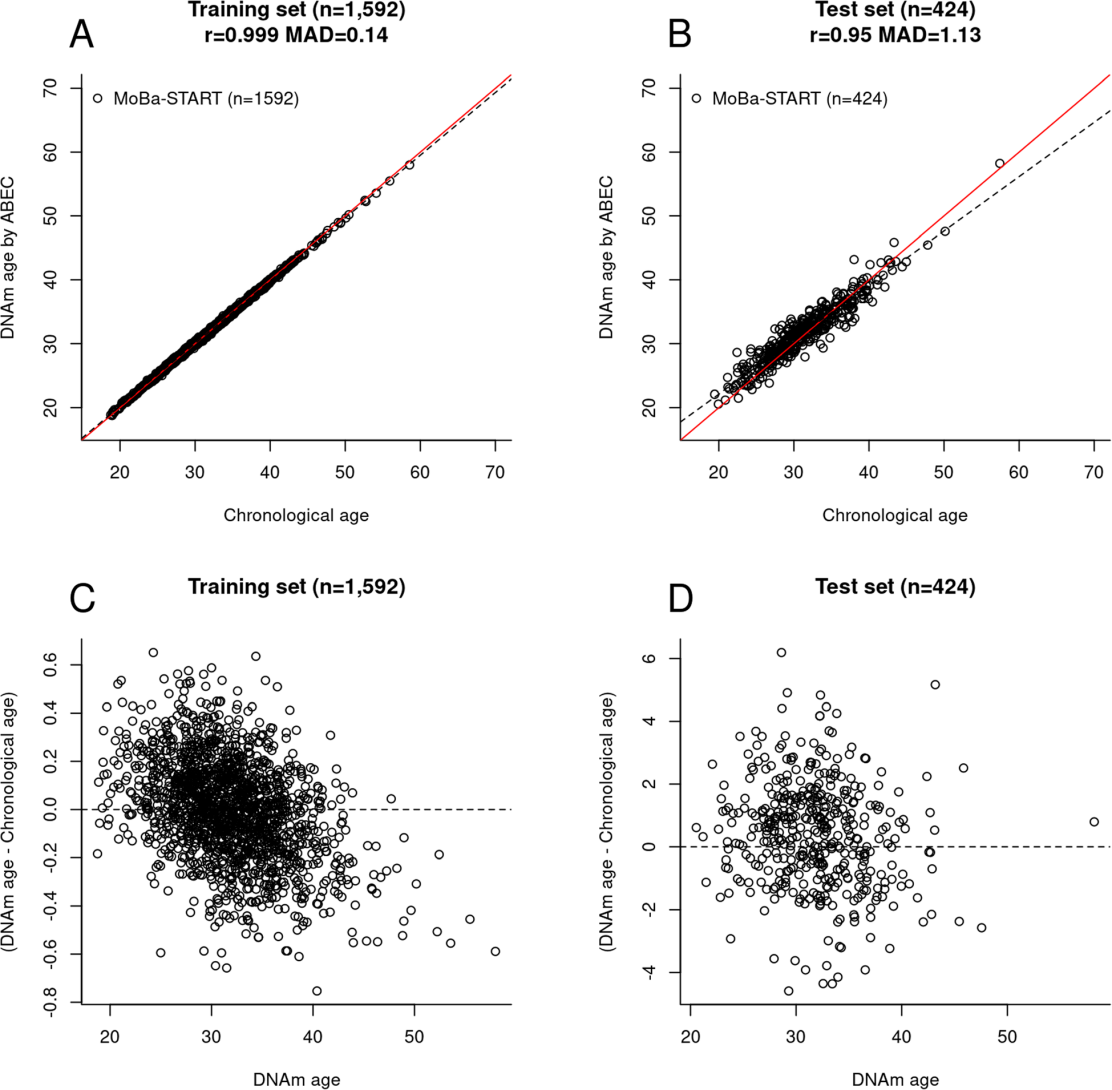
Chronological age estimation by ABEC. **a** Scatter plot of chronological age against DNAm age estimated by ABEC in the training set. **b** Scatter plot of chronological age against DNAm age estimated by ABEC in the test set. **c** Residual plot in the training set. **d** Residual plot in the test set. The red line in panels (**a**) and (**b**) represents a perfect correlation between chronological age and DNAm age, and the dotted line is the regression of DNAm age on chronological age

Figure 2 shows the performance of ABEC in the training set (*n* = 1592, Fig. 2a) and the test set (*n* = 424, Fig. 2b). The prediction precision was quantified using the Pearson correlation coefficient (r) between DNAm age and chronological age. The prediction accuracy was quantified using the median absolute deviation (MAD) between DNAm age and chronological age. ABEC showed high precision and accuracy in both of the training (r = 0.999, MAD = 0.14, Fig. 2a) and test set (r = 0.95, MAD = 1.13, Fig. 2b). The red line in Fig. 2a and b represents a perfect correlation between chronological age and DNAm age, and the dotted line refers to the regression of the predicted DNAm age on chronological age.

Despite its overall high precision, ABEC slightly underestimated the age of the older individuals, particularly those above 45 years of age (Fig. 2c, d). This bias is expected given that the MoBa-START dataset is a pregnancy cohort with few individuals older than 45 years. In addition, most individuals aged 45 years or older were males, which may introduce a sex-bias in the prediction of chronological age.

### Extended adult blood EPIC clock (eABEC)

**Fig. 3:**
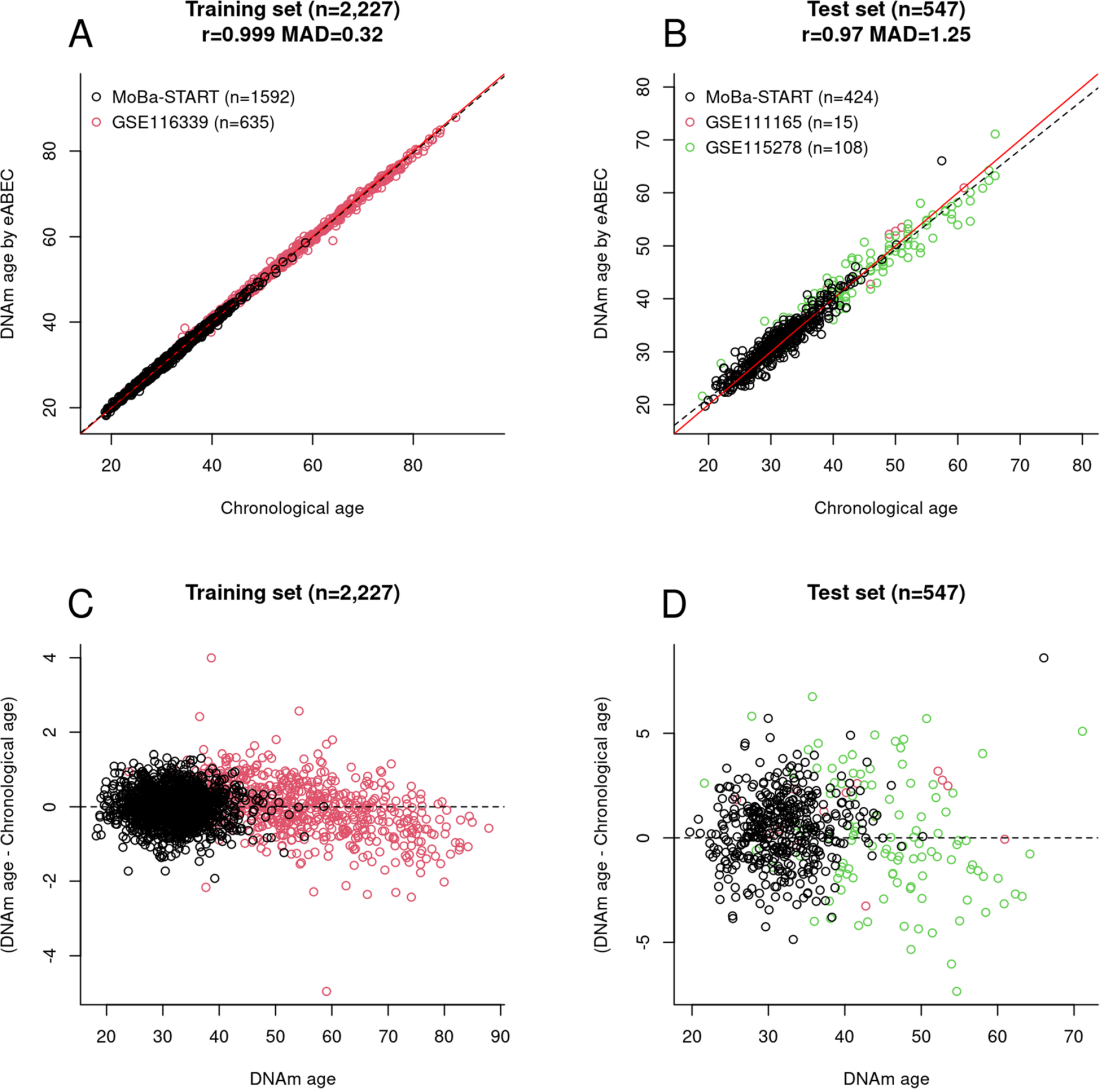
Chronological age estimation by eABEC. **a** Scatter plot of chronological age against DNAm age estimated by eABEC in the extensive training set. **b** Scatter plot of chronological age against DNAm age estimated by eABEC in the test set. **c** Residual plot in the training set. **d** Residual plot in the test set. The red line in panels (**a**) and (**b**) represents a perfect correlation between chronological age and DNAm age, and the dotted line is the regression of DNAm age on chronological age

To reduce the underestimation bias and improve the precision of ABEC among older individuals in the MoBa-START dataset, we developed an extended ABEC (eABEC) by adding a publicly available DNAm dataset, GSE116339 (*n* = 635) [25], from the GEO data repository (https://www.ncbi.nlm.nih.gov/geo/) [33] to the original training set for ABEC (Fig. 3). This increased the total sample size of the new training set to 2227. Elastic net regression was used in the same manner as for ABEC above, and for this training set, the number of selected CpGs was 1791.

We validated eABEC in an extended test set consisting of the test set for ABEC and two independent cohorts (GSE111165 and GSE115278) from GEO. We selected these GEO datasets because they were EPIC-derived blood-based DNAm data with a wide age span (20 to 70 years). The inclusion of GSE116339 substantially improved the prediction in individuals aged 45 years and above (Fig. 3a, b), but there was a slight underestimation of age among individuals aged 65 years or older in both the training and test set (Fig. 3c, d).

### Advantage of EPIC in developing epigenetic clocks

One major difference between our epigenetic clocks (ABEC and eABEC) and the previously published clocks was the use of EPIC for the training set. The training set of the other epigenetic clocks was mostly based on 450 K, except for the Horvath Skin & Blood clock which used both 450 K and EPIC-derived DNAm data. To assess whether EPIC-derived DNAm data yield a more accurate and precise clock, we trained a third epigenetic clock using the same training set as for eABEC but using only the 397,473 autosomal CpG sites that are in common between EPIC and 450 K. We refer to this third clock as ‘common’ ABEC (cABEC) hereafter. Elastic net regression selected 1892 CpG sites.

cABEC showed a high prediction performance, similar to eABEC (Supplementary File 2, S-Figure 1). The precision metric (r) of cABEC was identical to that of eABEC. However, compared to eABEC, the accuracy of cABEC in the test set was slightly diminished (MAD = 1.25 → 1.3).

**Fig. 4:**
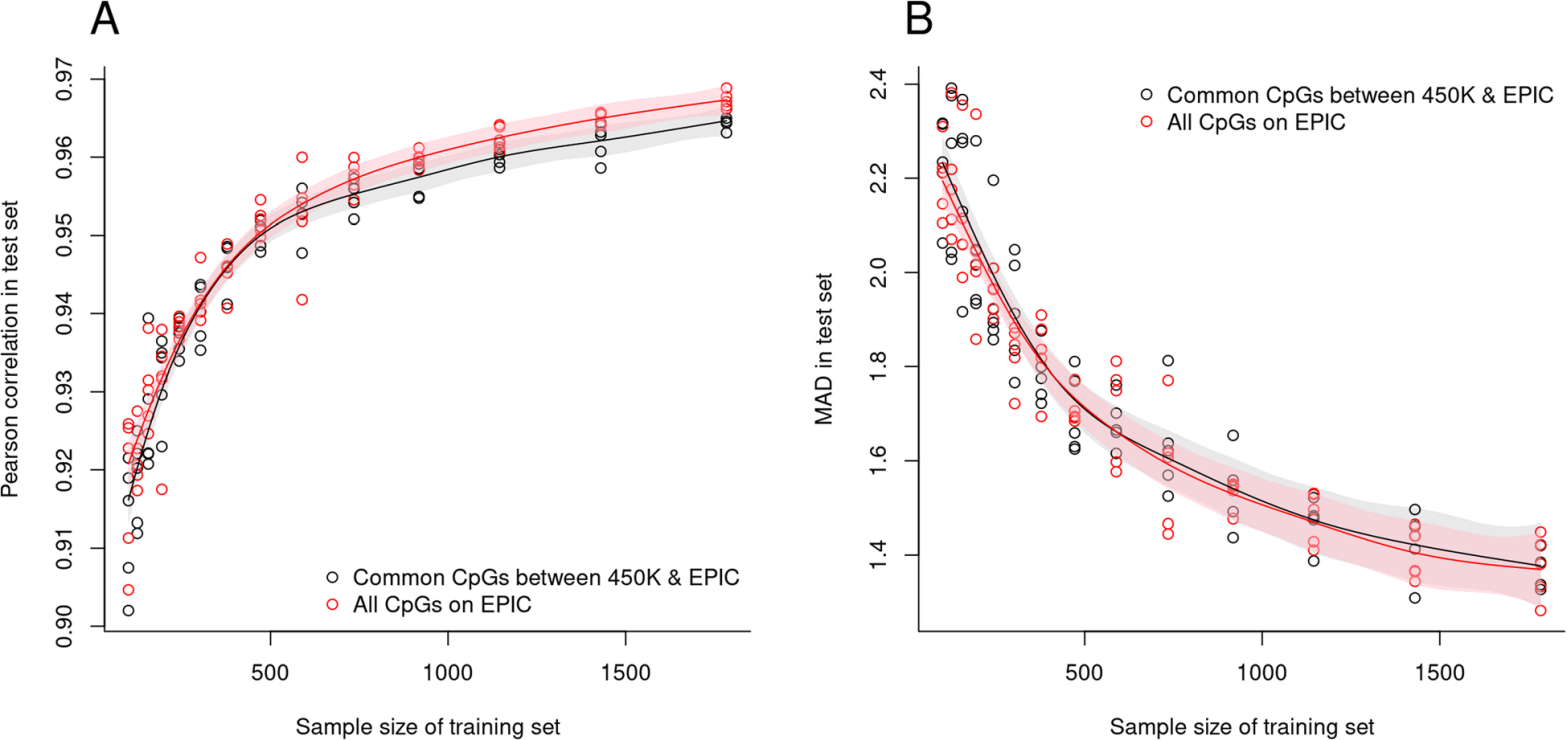
Comparison of precision and accuracy between a clock based on the CpGs common to 450 K and EPIC and a clock on all the CpGs on EPIC. **a** Scatter plot of the Pearson correlation (r) in the test set against the sample size of the training set. **b** Scatter plot of MAD in the test set against the sample size of the training set. In panel (**a**), we fit the smoothing splines of the Fisher’s Z-transformed r values on the sample size, derived the confidence intervals, and inverse-transformed them. In panel (**b**), we fit the smoothing splines of MAD values on the sample size without transformation. The black dots refer to the clock based on the CpGs common to 450 K and EPIC, and the red dots refer to the clock based on all the CpGs on EPIC

We hypothesized that the denser EPIC array might be beneficial in developing an epigenetic clock with a smaller training set. To address this point, two types of epigenetic clocks (one using all the CpGs on EPIC and the other using the CpGs common to EPIC and 450 K) were trained on random subsets of the training set of eABEC and validated in the test sample of eABEC (see Methods for further details). Both types of epigenetic clocks showed a remarkable improvement in precision and accuracy as the sample size of the training set increased (Fig. 4). However, across all the reduced training sets, the epigenetic clock based on all the CpGs on EPIC did not outperform the other clock based on the CpGs common to EPIC and 450 K (Fig. 4). This indicates that the additional CpGs on EPIC do not enhance the accuracy or precision of the epigenetic clocks when the training set is reduced.

### Validation of ABECs and other epigenetic clocks

Using an independent cohort from GEO (*n* = 123), we evaluated the performance of ABEC, eABEC, and cABEC against six published epigenetic clocks: the Hannum Blood-based clock [19], Horvath Pan-tissue clock [20], Levine PhenoAge clock [16], Horvath Skin & Blood clock [3], Alsaleh EPIC clock [22], and Zhang clock [34]. The independent test set consisted of GSE111165 [26] and GSE115278 [27] from the GEO database (see Table 1 for details). None of these GEO datasets have previously been used to train any epigenetic clocks.

**Fig. 5:**
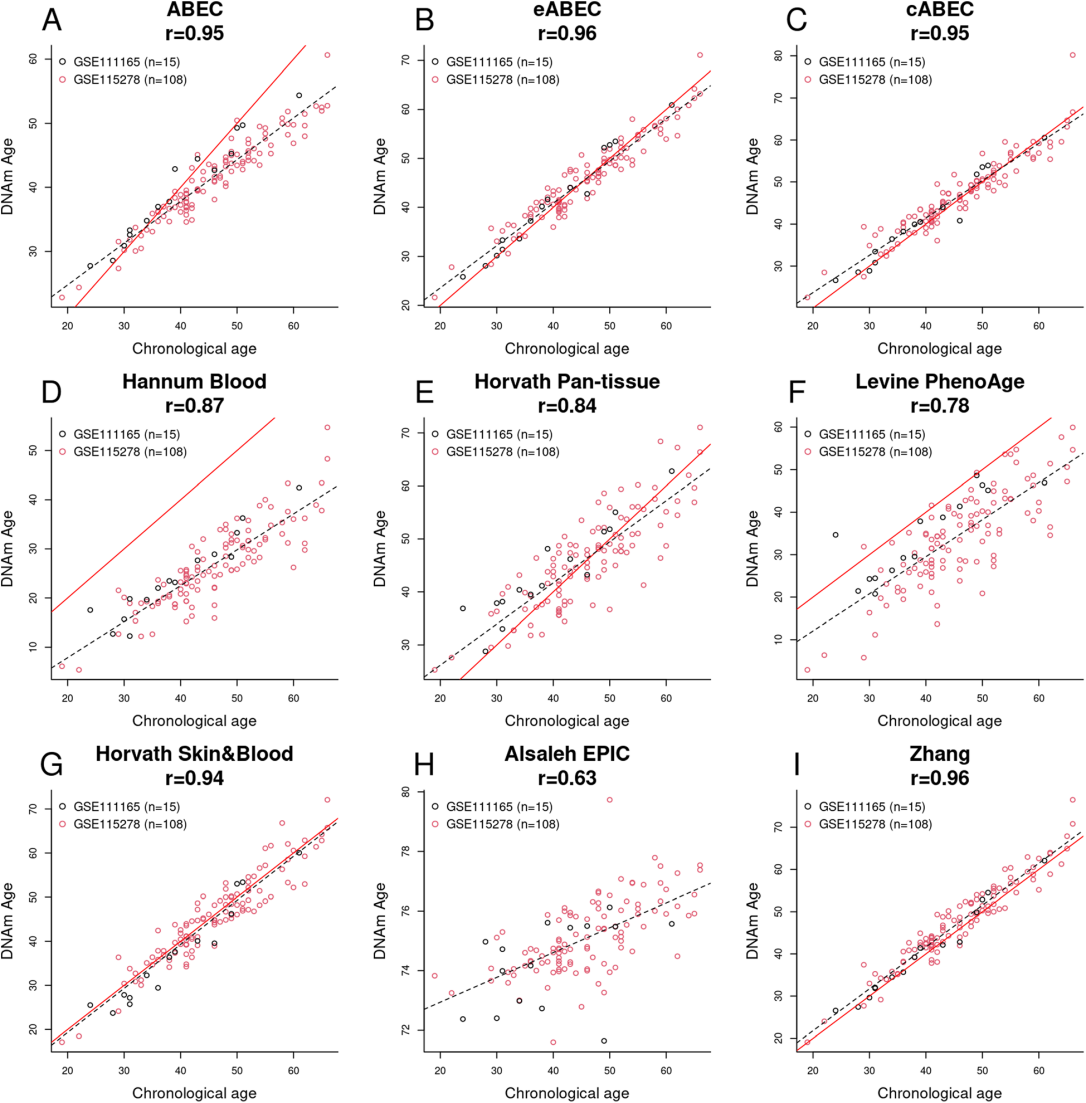
Chronological age estimation by ABEC, eABEC, and the other published epigenetic age estimators. **a** ABEC, **b** eABEC, **c** Hannum Blood-based clock, **d** Horvath Pan-tissue clock, **e** Levine PhenoAge clock, **f** Horvath Skin & blood clock, **g** Alsaleh Blood-based EPIC clock (the stepwise regression), and **h** Zhang clock (elastic net regression). The red line in the panels represents a perfect correlation between chronological age and DNAm age, and the dotted line is the regression of DNAm age on chronological age

Figure 5 summarizes the results of epigenetic age prediction by ABEC, eABEC, cABEC, and the six published epigenetic clocks mentioned above. Our eABEC and the Zhang clock showed the highest precision (r = 0.96), followed by ABEC (r = 0.95), cABEC (r = 0.95), the Horvath Skin & Blood clock (r = 0.94), and the Hannum Blood-based epigenetic clock (r = 0.87). The 95% confidence intervals of the r values can be found in Supplementary File 2 (S-Table 1). Here, we note that only the precision metric (r) was presented in Fig. 5 because the dots in the scatter plots could deviate systematically from the 45-degree line (so-called systematic offset) but still form a very tight prediction, e.g., panel (D) in Fig. 5. In such cases where high precision and relatively low accuracy are present, the systematic offset can be calibrated using a linear transformation, or, if necessary, a non-linear transformation.

**Fig. 6:**
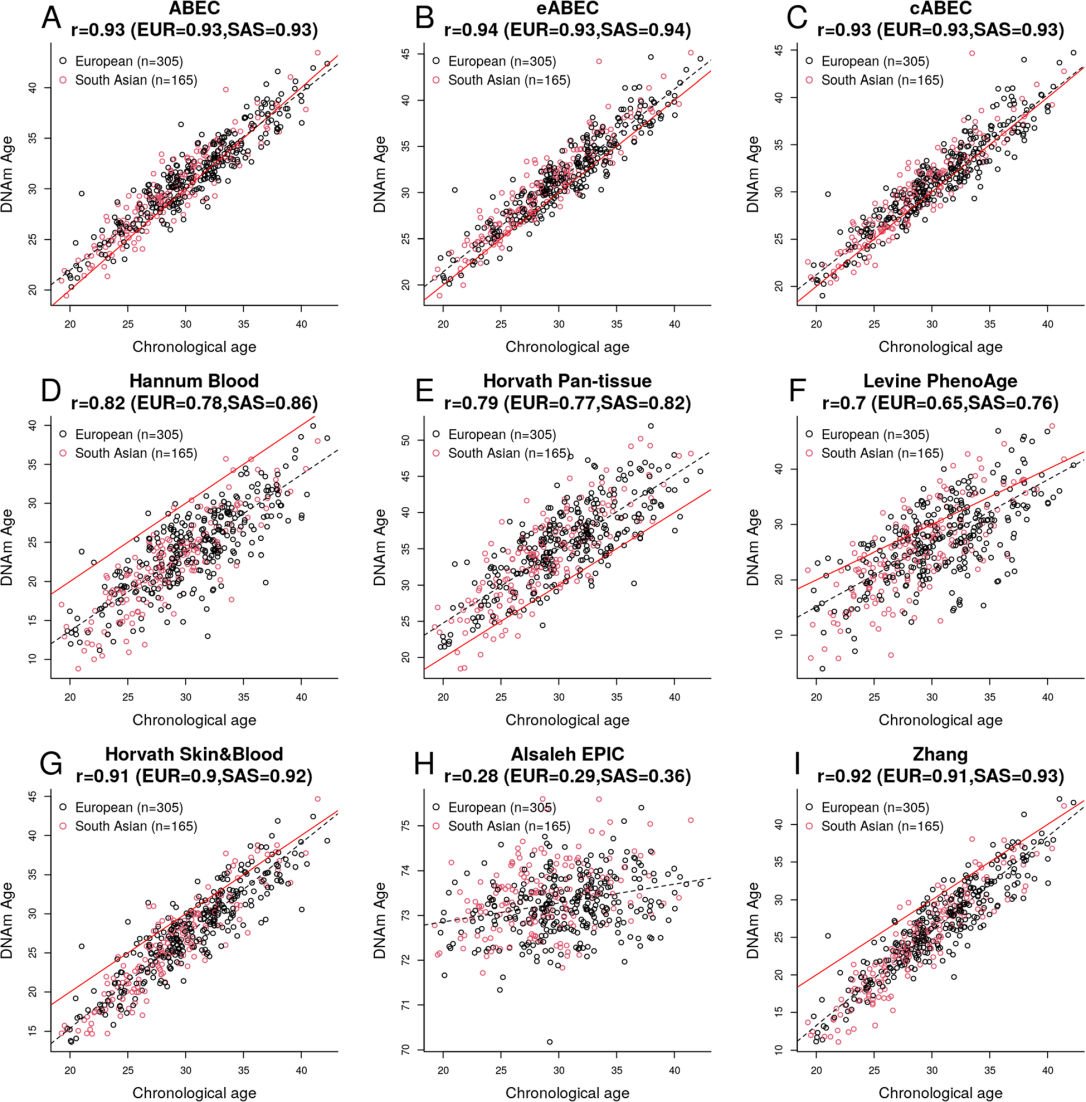
Application of ABEC, eABEC, and other epigenetic clocks to DNAm data in the EPIPREG sub-study of the STORK Groruddalen cohort. The title of each panel displays the overall r as well as the ethnicity-specific r. EUR indicates the r between chronological age and DNAm age in 305 women of European ancestry, whereas SAS refers to the r between chronological age and DNAm age in 165 women of South Asian ancestry

An important distinction of ABECs from the other published clocks is that they are based on an ethnically homogeneous training set (MoBa-START and GSE116339 comprised individuals of European ancestry). We validated ABEC, eABEC, cABEC, and the other published epigenetic clocks in the EPIC-derived blood-based DNAm data from EPIPREG (*n* = 470; 305 European women and 165 South Asian women, Fig. 6), a sub-study of the STORK Groruddalen Cohort [24]. ABEC, eABEC, cABEC, the Horvath Skin & Blood clock, and Zhang clock showed the highest precisions (r > 0.9). More interestingly, eABEC showed that the epigenetic age acceleration (EAA; residuals from the regression of DNAm age on chronological age) was higher in South Asian women than in Norwegian women (+ 0.51 years, *P* = 0.0015, Supplementary File 2, S-Figure 2A). EAA derived by the Alsaleh EPIC clock was also elevated in South Asians compared to Norwegians (+ 0.25 years, *P* = 4E-04, Supplementary File 2, S-Figure 2B). However, EAAs derived by ABEC, cABEC, and the other published clocks did not show any difference between the two groups.

Given that ABEC, eABEC, and cABEC were trained on the ethnically homogeneous training set of Europeans, they may be sub-optimal for predicting chronological age in other ethnicities. To explore this further, we applied ABEC, eABEC, cABEC, and the other published epigenetic clocks to a GEO dataset comprising African Americans (GSE132203 [28]; *n* = 795, Supplementary File 2, S-Figure 3). All the clocks, except for the Alsaleh EPIC clock, showed high correlations between chronological age and epigenetic age (r > 0.86). The 95% confidence intervals of the r values can be found in Supplementary File 2 (S-Table 1). eABEC, cABEC, and the Zhang clock showed the highest r of 0.96, and ABEC and the Horvath Skin & Blood clock showed the second-highest r of 0.95.

## Discussion

We developed precise epigenetic clocks (ABEC and eABEC) using blood-based DNAm data from EPIC. Our epigenetic clocks showed a more precise chronological age prediction than existing blood-based epigenetic clocks (e.g., the Hannum Blood-based clock and Horvath Skin & Blood clock; Fig. 5). The reason for the higher precision is more likely due to the large training set (*n* = 2227, Table 1) and the wide age-span of the samples (19 to 88 years for the training set of eABEC, Table 1), which is consistent with the findings by Zhang and colleagues [34]. Compared to eABEC, both Hannum Blood-based clock and Horvath Skin & Blood clock were trained on fewer samples (*n* = 656 and *n* = 896, respectively) that had a wider age-span (19 to 101 years and 0 to 94 years, respectively) [3, 19]. Other clocks (the Horvath Pan-tissue clock and Levine PhenoAge clock) may not be directly comparable to eABEC for chronological age prediction. For instance, the Horvath Pan-tissue clock was designed to measure epigenetic aging not only in blood but in multiple tissues [20], and the Levine PhenoAge was designed to predict phenotypic age (estimated using 10 clinical biomarkers, e.g., albumin, creatinine, serum glucose, and seven others) based on DNAm [16].

To develop eABEC, we added GSE116339 to the training set of ABEC. GSE116339 is from a study by Curtis et al. [25] that used EPIC to measure DNAm in peripheral blood samples collected from 658 individuals of European ancestry (638 non-Hispanic and 20 Hispanic) in Michigan, USA. These individuals had been exposed to the endocrine-disrupting chemical polybrominated biphenyl when an agricultural accident introduced it into the food supply in the 1970s. We selected 635 individuals from the control group whose total PBB (PBB-153, PBB-101, PBB-77, and PBB180) exposure was lower than 5 pg/ml. The distribution of the total PBB exposure was highly right-skewed.

The high precision of eABEC cannot be attributed solely to the use of the EPIC platform as the additional 413,743 CpGs on EPIC did not improve age prediction noticeably (Fig. 4). Although the 1791 CpGs selected by eABEC included 1084 CpGs that only exist on EPIC, eABEC did not outperform cABEC that used the CpGs common to 450 K and EPIC. This indicates that 226,915 probes (out of 413,743) that are designed to cover regulatory regions (DNase proximal/distal [35] and FANTOM5 [36]) did not increase the precision of the epigenetic clocks significantly [21]. Yet, Pidsley et al. [21] reported that probes on EPIC cover 58% of FANTOM5 enhancers, 7% of distal, and 27% of proximal ENCODE regulatory regions, suggesting that the coverage of regulatory regions is still low. Thus, it is difficult to dismiss the possibility that other regulatory CpGs not currently included on EPIC might improve age prediction.

Underestimation and overestimation of epigenetic clocks should be carefully assessed using residual plots instead of scatter plots. As we regressed chronological age on DNAm levels (chronological age = DNAm levels + error), a scatter plot that displays chronological age on the x-axis and DNAm age on the y-axis may lead to the misconception that DNAm age is overestimated in the oldest age group and underestimated in the youngest age group (Supplementary File, S-Figure 4). In contrast, residual plots that display DNAm age on the x-axis and residuals (DNAm age minus chronological age) on the y-axis would enable a fair evaluation of prediction models. The strength of the current scatter plots lies in the visualization of EAA (the residuals of the regression of DNAm age on chronological age; i.e., the vertical distance between each dot and the dotted line in Figs. 2 and 3).

Our clocks, particularly eABEC, showed a systematic underestimation in older subjects, as was the case with the Horvath Pan-tissue clock and Hannum Blood-based clock in GSE132203 [37]. The systematic underestimation may be corrected by 1) adding more DNAm data of older subjects to the training set or 2) calibrating epigenetic clocks using a non-linear transformation (e.g., piecewise cubic regression (with a knot at 70) or smoothing spline of chronological age on DNAm age). However, we could not add more EPIC-derived DNAm data from older subjects (preferably subjects of European ancestry aged 70 to 80 years) to the training set for eABEC. We note that the underestimation in older subjects can cause EAA to be dependent on chronological age. Therefore, for other researchers who are interested in the association between EAA and a given phenotype, we recommend redefining EAA (e.g., regressing DNAm age on chronological age using a piecewise cubic regression or a smoothing spline rather than an ordinary linear regression) so that EAA is independent of chronological age.

Our eABEC may result in subtle differences in EAA across different ethnic groups, e.g., Supplementary File 2, S-Figure 2A. A hypothesis explaining this bias is that the CpGs included in eABEC may be located near SNPs with a low minor allele frequency [38]. The SNPs may influence the DNAm level at the CpGs if the minor allele frequencies at the SNPs differ across ethnicities. To address this point, we added SNP annotations generated by Zhou et al. [38] and McCartney et al. [39] to Supplementary File 1.

## Conclusion

Three blood-based epigenetic clocks were developed to estimate adults’ chronological age using EPIC-derived DNAm data. The precision of these clocks was high (r > 0.94) when validated in independent cohorts. The high level of precision was not explained by the broader genomic coverage of EPIC (> 850,000 CpG sites) but rather by the large training set (*n* = 2227) with a wide age-span (19 to 88 years).

## Methods

### Study population

MoBa is a nationwide pregnancy cohort study in which approximately 95,000 mothers, 75,000 fathers, and 114,000 children were recruited from 1998 to 2008 across Norway [23]. The participants completed a series of questionnaires that are also linked to information from the Medical Birth Registry of Norway [23]. Peripheral whole-blood samples were collected from the mothers at the 17th week of gestation and at birth and from the fathers at the 17th week of gestation. Cord-blood samples were collected from newborns at birth [40, 41]. The precise chronological age in days at blood draw was calculated for the fathers and mothers. Further details on MoBa have been described in previous publications [23, 40–42]. We used data from a sub-study of MoBa (MoBa-START) with blood-based DNAm data on 2016 adults (mothers and fathers who were randomly selected among complete mother-father-newborn trios in MoBa).

GSE116339 is an epigenome-wide association study (EWAS) of polybrominated biphenyl in peripheral blood [25]. GSE111165 explored the difference in genome-wide DNAm between brain and peripheral tissues (buccal, saliva, and blood) from epilepsy patients [26]. GSE115278 is an EWAS of insulin resistance, obesity, and metabolic complications [27, 43–45]. GSE132203 examined the association between DNAm and psychiatric or stress-related symptoms [28].

EPIPREG is nested within the STORK Groruddalen Cohort study (a population-based cohort, *n* = 823, [24]). EPIPREG quantified DNAm in white blood cells, collected at the 28th week of gestation, from 480 women (312 of European ancestry and 168 of South Asian ancestry), using EPIC. In this study, we focused on 470 women (305 of European ancestry and 165 of South Asian ancestry) after excluding eight samples with low quality and two samples with an absolute EAA larger than 15 years. Further details of EPIPREG are described in Supplementary File 3 (S-Figure 7).

The age distributions of all the individuals included in the training and test sets can be found in Supplementary File 2 (S-Figure 5 and 6).

### Pre-processing of DNA methylation

For MoBa-START, 500 nanograms of DNA stored in the MoBa Biobank (see Paltiel et al. [41] for further details of the storage of the biological samples) were shipped to LIFE & BRAIN GmbH (Bonn, Germany). The samples were bisulfite converted and processed using the EZ-96DNA methylation-Lightning™MagPrep kit (Zymo Research, Irvine, USA) according to the manufacturer’s instructions. The raw iDAT files were imported and processed using the RnBeads R package [46]. 44,210 probes with cross-hybridization [39], high detection *p*-value (> 0.01), and 16,117 probes near single-nucleotide polymorphisms (filtering.snp = “3”) were excluded. The data were run in four batches and the exclusion criteria for removing probes were applied to each batch separately. Probes that were excluded from one batch were removed from all batches. The DNAm signals at the remaining probes were control-normalized and corrected for background noise using the *wm.nasen* and *methylumi.noob* options. Additionally, among a total of 2034 non-replicated samples, we excluded 18 samples that displayed low signal intensities and deviated (outliers) from the clusters formed by principal component analysis. The two probe chemistries (Type I and Type II probes) were normalized using Beta-mixture quantile normalization (BMIQ, [30]) using the wateRmelon R package [47]. In summary, the number of remaining probes was 790,213 (770,586 from autosomes and 19,627 from sex-chromosomes).

For the DNAm data from GEO, we downloaded the iDAT files and used normal-exponential out-of-band (Noob, [29]) normalization in the minfi R package [48]. For the DNAm data from EPIPREG, we performed functional normalization (FunNorm, [31]) using the meffil R package [49]. Further details of the DNA extraction and quality control process of EPIPREG can be found in Supplementary File 3.

### Elastic net regression

Penalized regressions (glmnet R package [50]) were used to develop the three ABECs. Chronological age in days was regressed on 770,586 autosomal CpGs that remained after quality control. The mixing parameter (alpha) was set to 0.5 and the shrinkage parameter (lambda) leading to the minimum mean square error was selected after 10-fold cross-validation in the training set. Supplementary File 3 (S-Figure 8) includes cross-validation curves for lambda and alpha values. ABEC, eABEC and cABEC selected 1695 CpG sites (lambda = 0.02884886), 1791 CpG sites (lambda = 0.05281471), and 1892 CpG sites (lambda = 0.0438477), respectively. Supplementary File 1 lists these CpG sites, their corresponding coefficients for ABEC, eABEC, and cABEC, and SNP annotations generated by Zhou et al. [38] and McCartney et al. [39].

### Comparison between EPIC-CpG clock and common CpG clock

The implementation resembles bootstrapping conceptually. For each of the reduced sample sizes (*n* = 100, 125, 156, 194, 243, 303, 378, 472, 589, 735, 918, 1145, 1430 and 1784; the determination of these values is detailed in Supplementary File 3), we first constructed five training sets by randomly selecting subjects from the full training set of eABEC (*n* = 2227). We made the sequence of the reduced sample sizes denser around 100 and sparser around 2227 because epigenetic clocks gradually improved their precision and accuracy when the training set was larger than 1145. On each training set, we trained two types of epigenetic clocks: one using all the CpGs on EPIC and the other using the CpGs common to EPIC and 450 K. Next, we validated these clocks in the test set of eABEC (*n* = 485) and calculated r and MAD accordingly. The mgcv R package [51] was used to fit the smoothing splines in Fig. 4. Particularly, in Fig. 4a, we fit the smoothing splines of the Fisher’s Z-transformed r values ($F(r)=0.5\ast \log \left(\frac{1+r}{1-r}\right)$) on the sample size, derived the confidence intervals and inverse-transformed them.

### Availability of epigenetic clocks

The estimated intercepts and coefficients for ABEC, eABEC, and cABEC can be found in Supplementary File 1.

The ABECs can be readily applied to any DNAm data using the following procedure: 1) generate a matrix of beta values (*n* individuals by *p* CpG sites) using a background correction method, e.g., Noob (preferably) without any batch adjustment (Supplementary File 3), 2) select the CpG sites for the ABECs (Supplementary File 1) out of the matrix of beta values, 3) calculate the linear combination of the beta values at the selected CpG sites, and 4) add the estimated intercept (Supplementary File 1) to the linear combination.

## Supplementary Information

**Additional file 1.** This file includes CpG sites for ABEC, eABEC, and cABEC, their corresponding coefficients, overlap with the other published clocks, genomic locations, neighboring genes, presence in the Illumina HumanMethylation450K and 27 K array, and the SNP annotations generated by Zhou et al. [38] (with the suffix of “Zhou”) and McCartney et al. [39] (with the suffix of “McCartney”).

**Additional file 2.** This file includes 1) a figure displaying the age prediction of cABEC, 2) a table containing the bootstrapped 95% confidence intervals for the r values in Figs. 4, 5 and 6) figures displaying the age prediction of the ABECs and the other published clocks in EPIPREG and GSE132203, 4) a figure illustrating the regression-to-the-mean effect and 5) histograms displaying the age distribution of individuals in each cohort.

**Additional file 3.** This file includes 1) further details (sample selection, DNA extraction, and quality control) of EPIPREG, 2) cross-validation curves of mean squared error over lambda and alpha values for eABEC, 3) determination of the reduced sample sizes for Fig. 4, and 4) further information regarding batch adjustment in developing the ABECs.

## Data Availability

The MoBa data can be accessed by applying directly to the Norwegian Institute of Public Health, http://www.fhi.no/en/. The EPIPREG data can be accessed by contacting Dr. Christine Sommer, Oslo University Hospital, https://www.oslodiabetes.no/christine-sommer. The publicly available DNAm data in this study (accession numbers: GSE116339, GSE111165, GSE115278, and GSE132203) are accessible on the GEO repository, https://www.ncbi.nlm.nih.gov/geo/. Public access to the GEO repository is open, and thus administrative permission to access and use the data is not needed.

## Abbreviations

DNAm: DNA methylation
ABEC: Adult Blood EPIC Clock
eABEC: extended Adult Blood EPIC Clock
cABEC: common Adult Blood EPIC Clock
GEO: Gene Expression Omnibus
EPIC: Illumina MethylationEPIC BeadChip
450 K: Illumina HumanMethylation 450 K BeadChip
r: The Pearson correlation coefficient
MAD: Median absolute deviation
BMIQ: Beta-mixture quantile dilation
MoBa: Norwegian Mother, Father and Child Cohort Study
START: Study of Assisted Reproductive Technology
EPIPREG: Epigenetics in Pregnancy
EAA: Epigenetic age acceleration
