# Supplementary material for: Blood-based epigenetic estimators of chronological age in human adults using DNA methylation data from the Illumina MethylationEPIC array
Source: BMC Genomics. 2020 Oct 27;21:747. doi: 10.1186/s12864-020-07168-8 (PMC7590728; doi:10.1186/s12864-020-07168-8)
Supplement: Supplementary file 2 — Additional file 2. This file includes 1) a figure displaying the age prediction of cABEC, 2) a table containing the bootstrapped 95% confidence intervals for the r values in Figs. 4, 5 and 6) figures displaying the age prediction of the ABECs and the other published clocks in EPIPREG and GSE132203, 4) a figure illustrating the regression-to-the-mean effect and 5) histograms displaying the age distribution of individuals in each cohort. [file 12864_2020_7168_MOESM2_ESM.docx]

**Supplementary File 2**

**
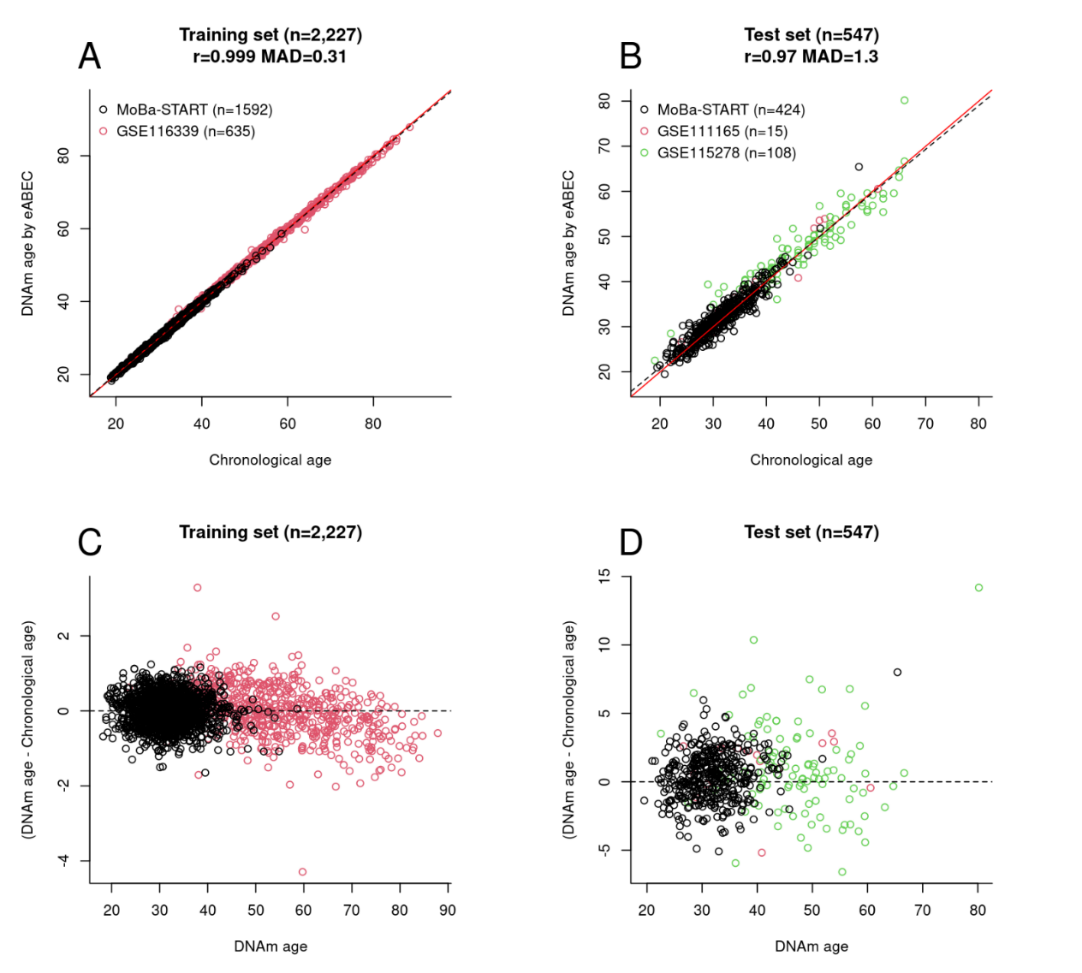
**

**S-Figure 1. Chronological age prediction of cABEC based on 397,473 autosomal CpG sites that exist on both EPIC and 450K.**

(A) Scatter plot of chronological age against DNAm age estimated by ABEC in the training set. (B) Scatter plot of chronological age against DNAm age estimated by ABEC in the test set. (C) Residual plot in the training set. (D) Residual plot in the test set.

The red line in panels (A) and (B) represents a perfect correlation between chronological age and DNAm age, and the dotted line is the regression of DNAm age on chronological age.

**S-Table 1. Bootstrapped confidence intervals of the precision metric (r) values in Figure 5 and 6.**

| Type |  | r | 95% CI  lower limit | 95% CI  upper limit |
| --- | --- | --- | --- | --- |
| **Figure 5** | |  |  |  |
|  | ABEC | 0.9491 | 0.9287 | 0.9648 |
|  | eABEC | 0.9625 | 0.949 | 0.9733 |
|  | cABEC | 0.9483 | 0.9282 | 0.9675 |
|  | Hannum | 0.8693 | 0.8239 | 0.9061 |
|  | Horvath Pan-Tissue | 0.8421 | 0.7862 | 0.8858 |
|  | Levine PhenoAge | 0.7829 | 0.6981 | 0.8498 |
|  | Horvath Skin&Blood | 0.9432 | 0.9218 | 0.9599 |
|  | Alsaleh EPIC | 0.6269 | 0.5168 | 0.7312 |
|  | Zhang | 0.9641 | 0.9506 | 0.9748 |
|  |  |  |  |  |
| **Figure 6 - ALL** | |  |  |  |
|  | ABEC | 0.9313 | 0.9169 | 0.9436 |
|  | eABEC | 0.9351 | 0.9206 | 0.9474 |
|  | cABEC | 0.9309 | 0.9148 | 0.9442 |
|  | Hannum | 0.8155 | 0.7799 | 0.8462 |
|  | Horvath Pan-Tissue | 0.7908 | 0.7559 | 0.8217 |
|  | Levine PhenoAge | 0.6961 | 0.6456 | 0.7404 |
|  | Horvath Skin&Blood | 0.9096 | 0.8911 | 0.9254 |
|  | Alsaleh EPIC | 0.2808 | 0.1984 | 0.3593 |
|  | Zhang | 0.919 | 0.9022 | 0.9333 |
|  |  |  |  |  |
| **Figure 6 - EUR** | |  |  |  |
|  | ABEC | 0.9292 | 0.9095 | 0.9452 |
|  | eABEC | 0.9343 | 0.9162 | 0.9488 |
|  | cABEC | 0.929 | 0.9103 | 0.9442 |
|  | Hannum | 0.7832 | 0.7302 | 0.8277 |
|  | Horvath Pan-Tissue | 0.7671 | 0.7149 | 0.8109 |
|  | Levine PhenoAge | 0.6506 | 0.5791 | 0.712 |
|  | Horvath Skin&Blood | 0.9006 | 0.8747 | 0.9223 |
|  | Alsaleh EPIC | 0.2899 | 0.1874 | 0.3865 |
|  | Zhang | 0.9108 | 0.8862 | 0.9307 |
|  |  |  |  |  |
| **Figure 6 - SOU** | |  |  |  |
|  | ABEC | 0.9292 | 0.9051 | 0.9484 |
|  | eABEC | 0.939 | 0.9153 | 0.9579 |
|  | cABEC | 0.9318 | 0.9047 | 0.9537 |
|  | Hannum | 0.8559 | 0.8077 | 0.8939 |
|  | Horvath Pan-Tissue | 0.8164 | 0.763 | 0.8592 |
|  | Levine PhenoAge | 0.7617 | 0.6917 | 0.8196 |
|  | Horvath Skin&Blood | 0.9184 | 0.8895 | 0.9408 |
|  | Alsaleh EPIC | 0.3605 | 0.2277 | 0.4827 |
|  | Zhang | 0.9274 | 0.9047 | 0.9463 |

**
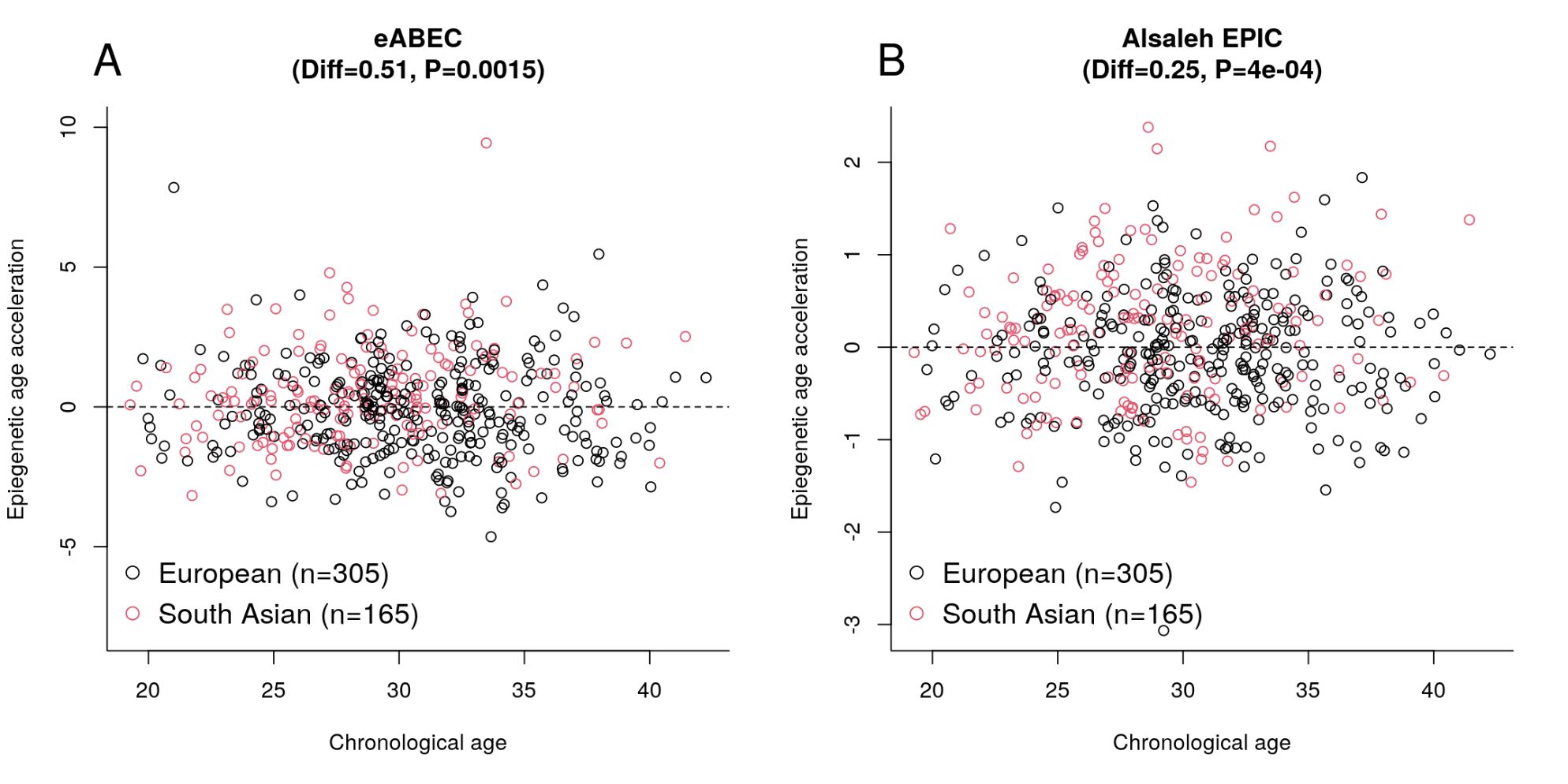
**

**S-Figure 2. Scatter plots of epigenetic age acceleration against chronological age in EPIPREG.**

(A) EAA was calculated from DNAm age estimated by eABEC. (B) EAA was calculated from DNAm age estimated by the Alsaleh EPIC clock.

Epigenetic age acceleration (EAA) is the residual of a robust MM-type regression of DNAm age on chronological age. Diff refers to the difference in EAA (in years) between European and South Asian women, and P refers to the corresponding p-value of the difference.


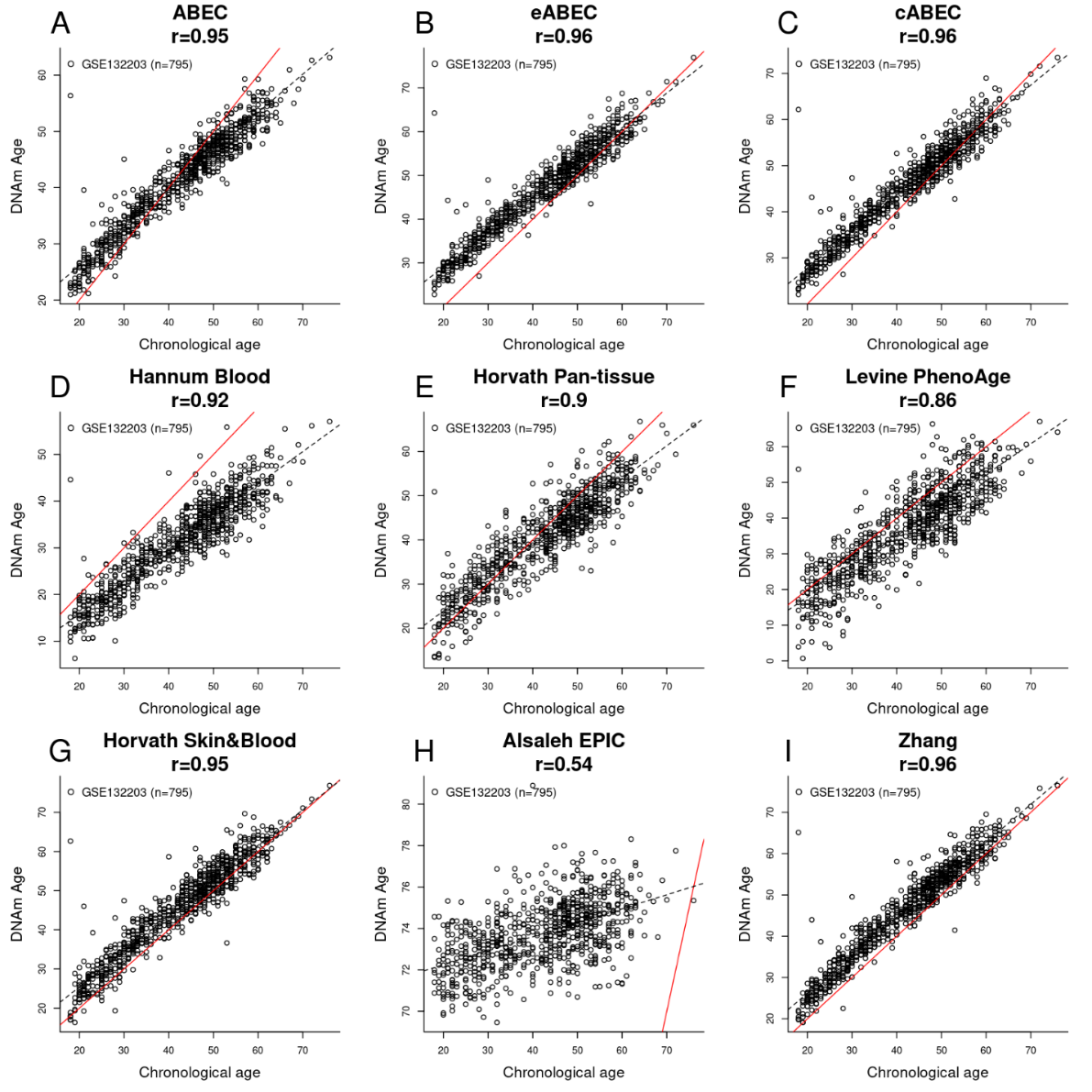


**S-Figure 3. Application of ABEC, eABEC, cABEC, and six other epigenetic clocks to GSE132203 (n=795, African Americans).**

The red line in the panels represents a perfect correlation between chronological age and DNAm age, and the dotted line refers to the regression of DNAm age on chronological age. The Zhang clock in this figure refers to the elastic net model.


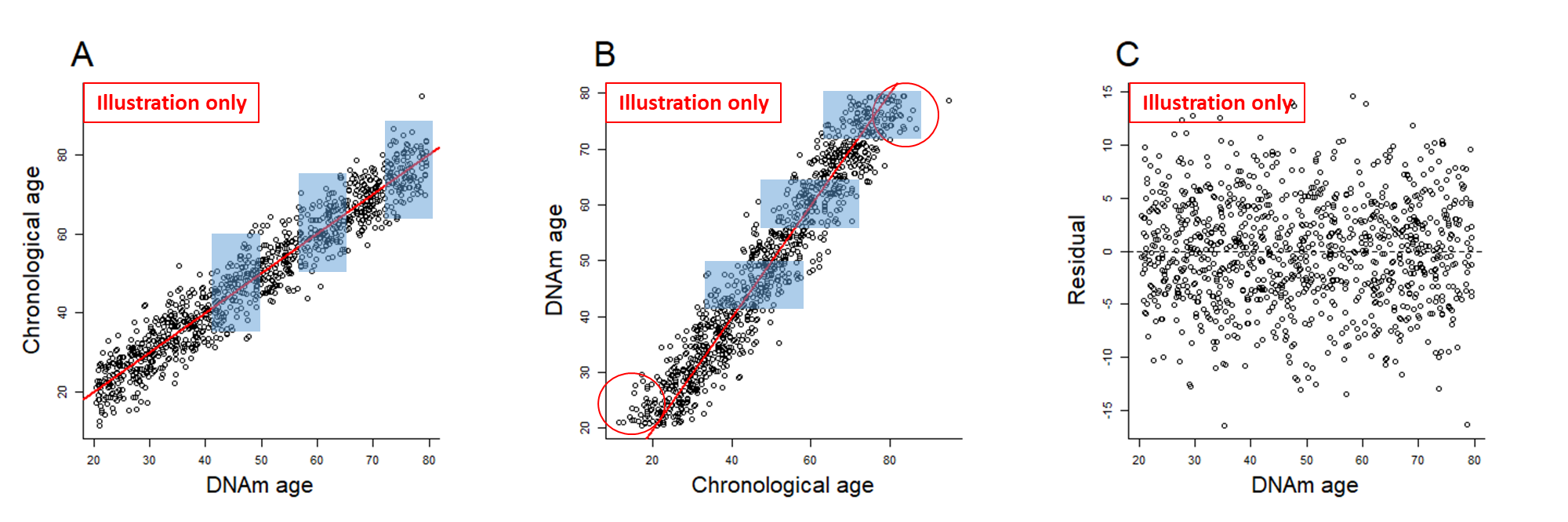


**S-Figure 4. Visualization of the regression-to-the-mean effect in prediction models.**

(A) Scatter plot of chronological age against DNAm age. (B) Scatter plot of DNAm age against chronological age. (C) Residual plot.

All the panels have been created for illustrative purposes only. The prediction model (epigenetic clock) of chronological age on DNAm levels was developed without any under or overestimation. However, the setting in panel (B) gives the erroneous impression of an underestimation in older subjects and an overestimation in younger subjects. This is due to the regression-to-the-mean effect (we regressed “chronological age” on DNAm levels; $chronological age=DNAm age+error$). The impression of under and overestimation do not appear in the residual plot in panel (C).


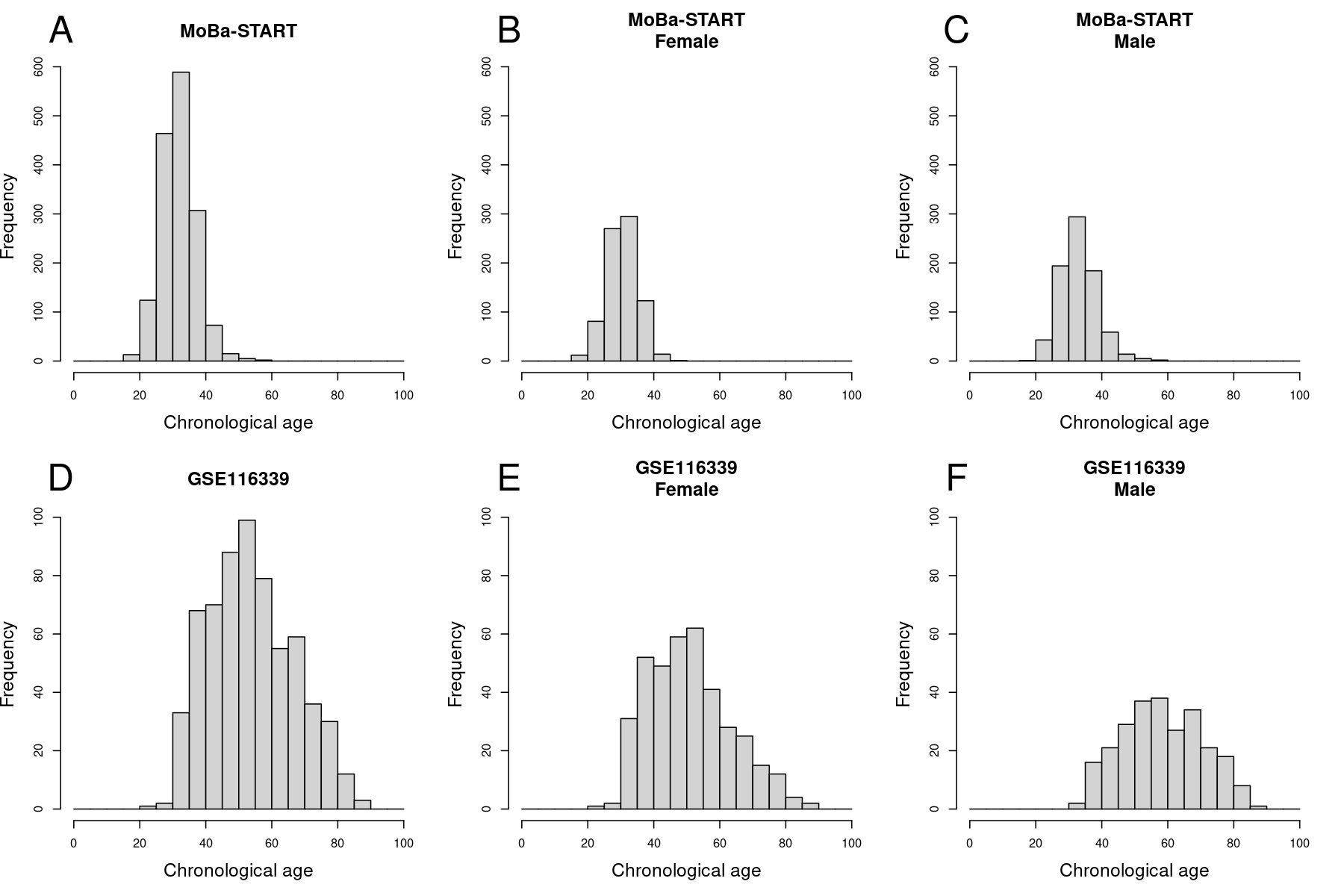


**S-Figure 5. Distribution of chronological age in the training set for eABEC.**

(A) – (C): MoBa-START and (D) – (F): GSE116339.

**
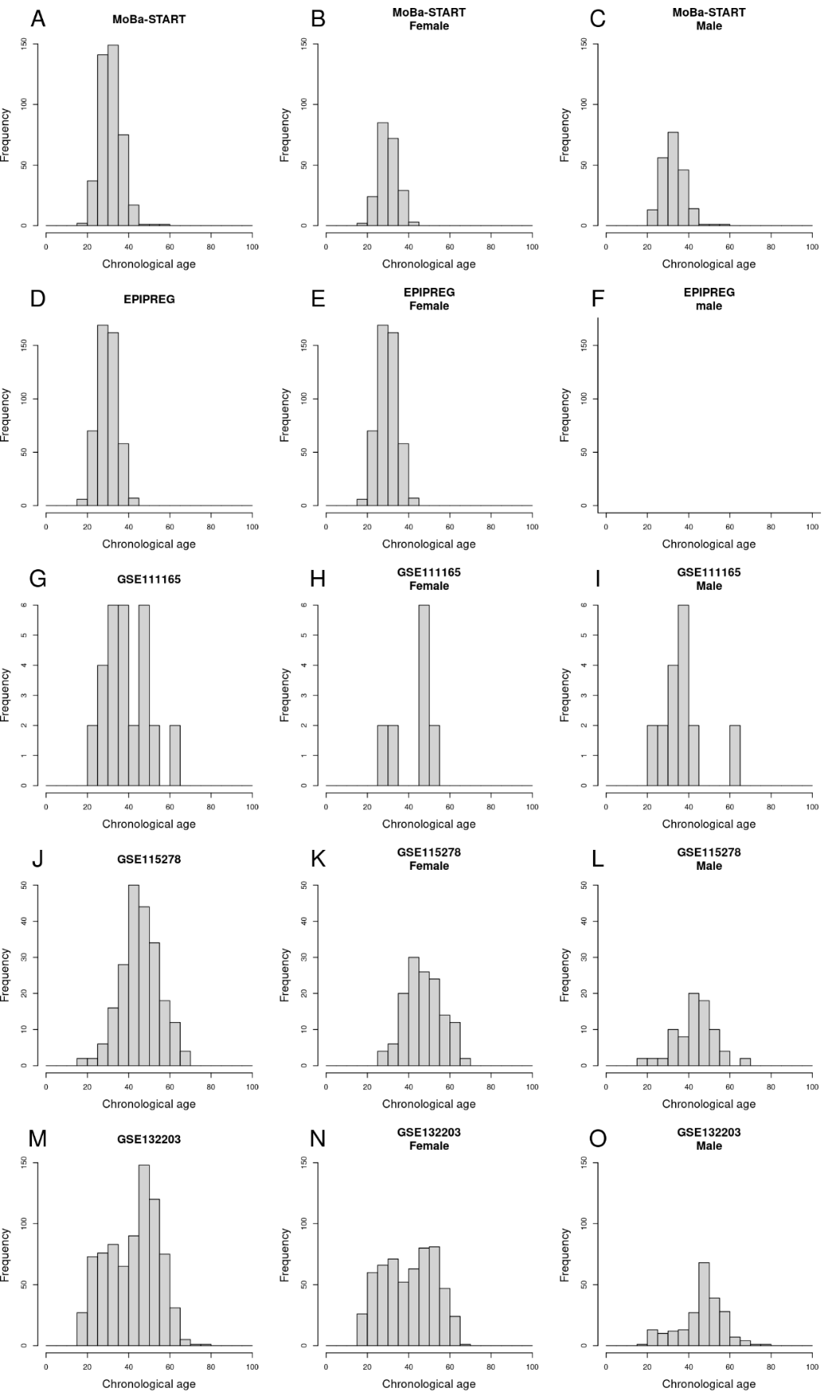
**

**S-Figure 6. Distribution of chronological age in the test sets.**

(A) – (C): MoBa-START, (D) – (F): EPIPREG, (G) – (I): GSE111165, (J) – (L): GSE115278, and (M) – (O): GSE132203.
