# Supplementary material for: Blood-based epigenetic estimators of chronological age in human adults using DNA methylation data from the Illumina MethylationEPIC array
Source: BMC Genomics. 2020 Oct 27;21:747. doi: 10.1186/s12864-020-07168-8 (PMC7590728; doi:10.1186/s12864-020-07168-8)
Supplement: Supplementary file 3 — Additional file 3. This file includes 1) further details (sample selection, DNA extraction, and quality control) of EPIPREG, 2) cross-validation curves of mean squared error over lambda and alpha values for eABEC, 3) determination of the reduced sample sizes for Fig. 4, and 4) further information regarding batch adjustment in developing the ABECs. [file 12864_2020_7168_MOESM3_ESM.docx]

**EPIPREG from STORK Groruddalen**

*Study population*

STORK Groruddalen is a population-based cohort of 823 healthy women attending three public mother–child health clinics for antenatal care in the multi-ethnic area of Groruddalen, Oslo, Norway, as described in detail previously [1]. Briefly, women were eligible if they 1) lived in the study districts, 2) planned to give birth at one of two study hospitals, 3) were <20 weeks pregnant, 4) could communicate in Norwegian or any of the eight translated languages, and 5) were able to give informed consent. Women with pre-gestational diabetes or in need of intensive hospital follow-up during pregnancy were excluded. The participation rate was 74%, varying from 63.9% to 82.6% across ethnic groups [1].

The cohort study, including its sub-studies on genetics and epigenetics, was approved by the Regional Committees for Medical and Health Research Ethics South-East (2015/1035) in Norway. We obtained written informed consent from each participant.


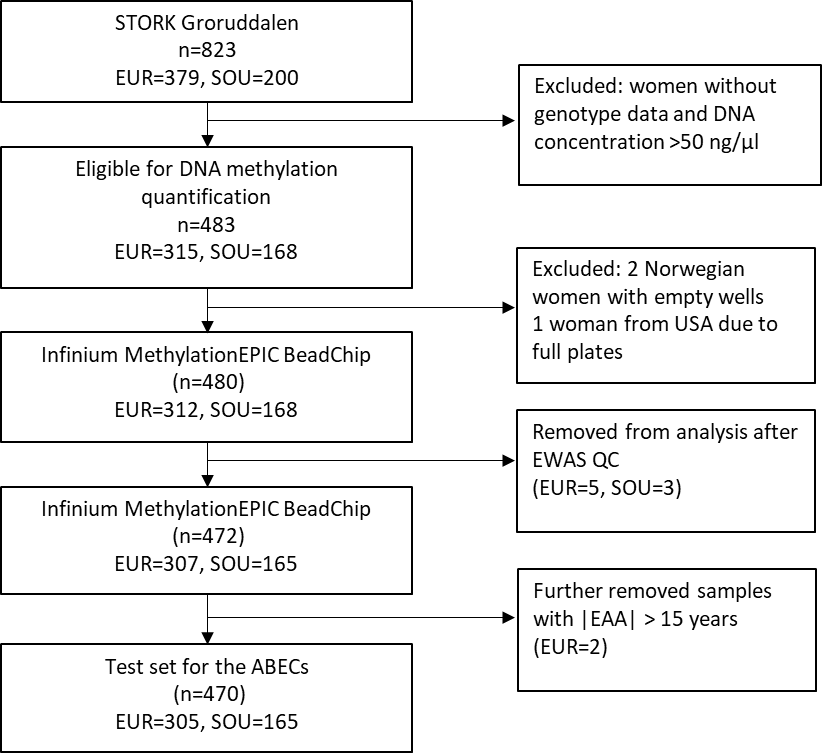


**S-Figure 7. Flow chart of the EPIPREG study sample.**

*EPIPREG*

For the Epigenetics in Pregnancy (EPIPREG) sub-study, we quantified DNA methylation in Europeans and South Asians participants in the STORK Groruddalen cohort (S-Figure 7). Participants without genotype data [2] and decent DNA quality (>50 ng/µl) were excluded. To define European and South Asian ancestry, principal component analysis based on the variance-standardized relationship matrix generated in the PLINK 1.9 software [3] (<https://www.cog-genomics.org/plink/1.9/>) was used. The analysis flow of the EPIPREG sample is shown in S-Figure 7. For Europeans, we measured DNA methylation in 99 % of the eligible participants (empty wells=2 and full plates=1), and 82 % of the total samples of Europeans included in the STORK Groruddalen cohort. For South Asians, we measured DNA methylation in 100 % of the eligible participants and 84 % of the total samples of South Asians included in the STORK Groruddalen cohort (S-Figure 7).

*DNA extraction*

In gestational week 28, maternal blood was drawn into tubes with ethylenediaminetetraacetic acid (EDTA), which were tilted but not centrifuged. DNA was extracted continuously throughout the data collection step, at the Hormone Laboratory, Oslo University Hospital, using a salting out procedure [4]. The resulting DNA was frozen on site and stored at -80°C.

*DNA methylation*

DNA samples were bisulfite-converted using the EZ DNA MethylationTM Kit (Zymo Research, Tustin, CA, USA) before they were added to the Infinium MethylationEPIC Kit (Illumina, San Diego, CA, USA) at the Department of Clinical Sciences, Clinical Research Centre, Lund University, Malmö, Sweden. Raw signal intensities of each probe were extracted using Illumina’s GenomeStudio Software.

*Quality control*

The methylation level at each site was represented as a beta (β) value of the fluorescence intensity ratio which ranged from 0 (not methylated) to 1 (completely methylated). The Meffil [5] R package (<https://cran.r-project.org/>) was used for quality control, normalization, and quantification of beta values. We removed eight samples (six with sex mismatch and two with median methylated/unmethylated comparison^[[1]](#footnote-1)^) and 1,299 probes with detection p-value <0.01, bead count <3, sex mismatch >5 SD or genotype mismatch. We used functional normalization, adjusting for the effects of different batches, plates, columns, and rows. A total of 472 individuals and 864,560 probes remained for the current analyses.

**Cross-validation curves of the mean squared errors over lambda and alpha values for eABEC.**

We undertook a 10-fold cross validation using a sequence of lambda values and the following alpha values: 0.1, 0.3, 0.7, and 0.9 (S-Figure 8). The differences among the minimum mean-squared errors for each alpha value were minimal, and their confidence intervals largely overlapped with one another. Given that the alpha values did not have a noticeable impact on the prediction performance overall, we decided to use an alpha of 0.5, which has consistently been used in other published clocks, e.g., the Horvath Pan-tissue [6], Horvath Skin Blood [7], and Zhang et al. [8].


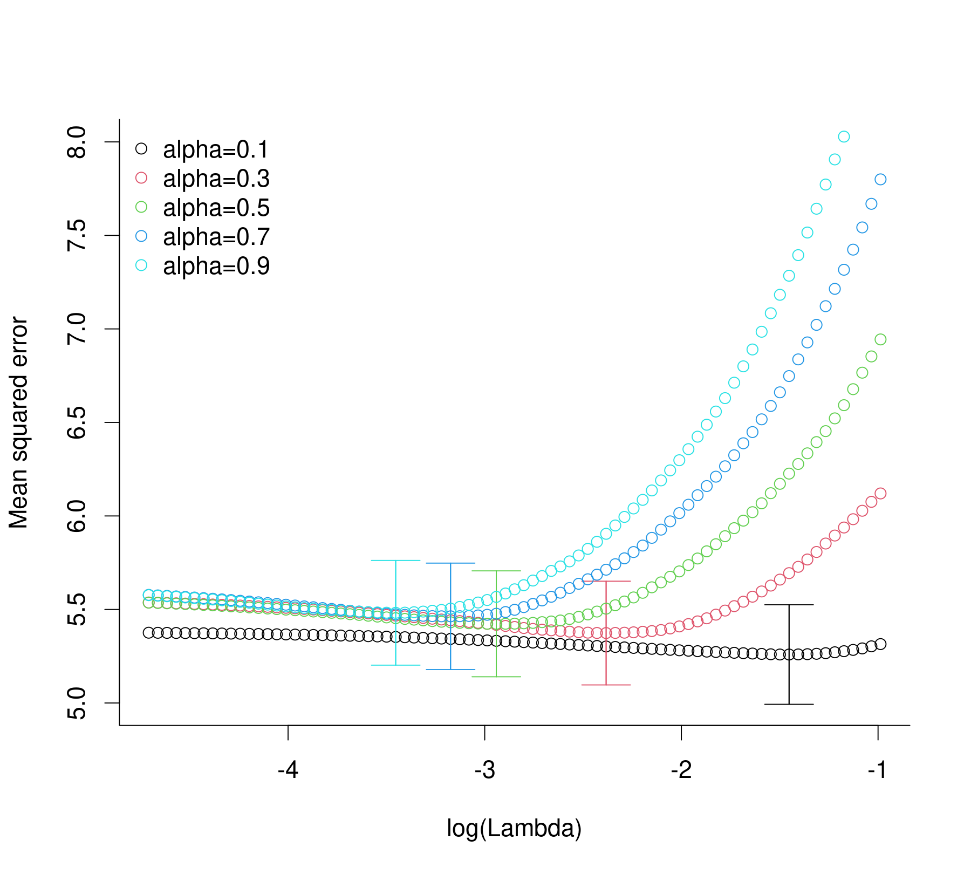


**S-Figure 8. Mean squared error of 10-fold cross validation for elastic net regressions with different mixing parameters.**

The 95% confidence intervals of the minimum mean squared error are displayed as vertical bars.

**Determination of the sample sizes for Figure 4**

We exponentiated the values between 100 and 2,227 using the following R code:

round(exp(seq(log(100),log(2227),length.out=15)))

The last term, 2,227, was excluded from the set of values because the resulting epigenetic clock based on the training set of 2,227 is identical to eABEC.

The epigenetic clocks performed well when their training sets reached a sufficiently large size. Here, we wanted to focus more on the performance of the epigenetic clocks trained on smaller training sets.

**Adjustment for batch effects**

We used the raw beta values for training the ABECs. Therefore, we expected the ABECs to be robust against potential batch effects in test sets. The ABECs showed high precision and accuracy in the test sets, which were not corrected for batch effects but for background noise (Figure 5 and S-Figure 3). This strategy is in line with the user guide for the Horvath online calculator (https://horvath.genetics.ucla.edu/html/dnamage/faq.htm), which recommends users not to remove batch effects or implement a complex normalization.

**References**

1. Jenum AK, Sletner L, Voldner N, Vangen S, Morkrid K, Andersen LF, Nakstad B, Skrivarhaug T, Rognerud-Jensen OH, Roald B *et al*: **The STORK Groruddalen research programme: A population-based cohort study of gestational diabetes, physical activity, and obesity in pregnancy in a multiethnic population. Rationale, methods, study population, and participation rates**. *Scand J Public Health* 2010, **38**(5 Suppl):60-70.

2. Arora GP, Akerlund M, Brons C, Moen GH, Wasenius NS, Sommer C, Jenum AK, Almgren P, Thaman RG, Orho-Melander M *et al*: **Phenotypic and genotypic differences between Indian and Scandinavian women with gestational diabetes mellitus**. *J Intern Med* 2019, **286**(2):192-206.

3. Purcell S, Neale B, Todd-Brown K, Thomas L, Ferreira Manuel A R, Bender D, Maller J, Sklar P, de Bakker Paul I W, Daly Mark J *et al*: **PLINK: A Tool Set for Whole-Genome Association and Population-Based Linkage Analyses**. *American Journal of Human Genetics* 2007, **81**(3):559-575.

4. Miller SA, Dykes DD, Polesky HF: **A simple salting out procedure for extracting DNA from human nucleated cells**. *Nucleic Acids Research* 1988, **16**(3):1215.

5. Min JL, Hemani G, Davey Smith G, Relton C, Suderman M: **Meffil: efficient normalization and analysis of very large DNA methylation datasets**. *Bioinformatics (Oxford, England)* 2018.

6. Horvath S: **DNA methylation age of human tissues and cell types**. *Genome Biol* 2013, **14**(10):R115.

7. Horvath S, Oshima J, Martin GM, Lu AT, Quach A, Cohen H, Felton S, Matsuyama M, Lowe D, Kabacik S *et al*: **Epigenetic clock for skin and blood cells applied to Hutchinson Gilford Progeria Syndrome and ex vivo studies**. *Aging (Albany NY)* 2018, **10**(7):1758-1775.

8. Zhang Q, Vallerga CL, Walker RM, Lin T, Henders AK, Montgomery GW, He J, Fan D, Fowdar J, Kennedy M *et al*: **Improved precision of epigenetic clock estimates across tissues and its implication for biological ageing**. *Genome Med* 2019, **11**(1):54.

1. The algorithm for detecting outliers was as follows: 1) derived a median methylated and a median unmethylated signal for each individual, 2) regressed the median methylated on the median unmethylated signal, and 3) detected outliers with residuals resulting from the regression in the earlier step being larger than 3 standard deviations. [↑](#footnote-ref-1)
